# NF‐κB/IKK activation by small extracellular vesicles within the SASP

**DOI:** 10.1111/acel.13426

**Published:** 2021-06-29

**Authors:** Juan Antonio Fafián‐Labora, Ana O’Loghlen

**Affiliations:** ^1^ Epigenetics & Cellular Senescence Group Blizard Institute Barts and The London School of Medicine and Dentistry Queen Mary University of London London UK; ^2^ Present address: Grupo de investigación en Terapia Celular y Medicina Regenerativa, Departamento de Fisioterapia, Medicina y Ciencias Biomédicas, Facultad de Ciencias de la Salud Universidade da Coruña, INIBIC‐Complejo Hospitalario Universitario A Coruña (CHUAC), Agrupación estratégica CICA‐INIBIC A Coruña Spain

**Keywords:** ageing, extracellular vesicles (EV), IκB kinase (IKK), NF‐κB, SASP, senescence

## Abstract

Cellular senescence plays an important role in different biological and pathological conditions. Senescent cells communicate with their microenvironment through a plethora of soluble factors, metalloproteases and extracellular vesicles (EV). Although much is known about the role that soluble factors play in senescence, the downstream signalling pathways activated by EV in senescence is unknown. To address this, we performed a small molecule inhibitor screen and have identified the IκB kinases IKKε, IKKα and IKKβ as essential for senescence mediated by EV (evSASP). By using pharmacological inhibitors of IKKε, IKKα and IKKβ, in addition to CRISPR/Cas9 targeting their respective genes, we find these pathways are important in mediating senescence. In addition, we find that senescence activation is dependent on canonical NF‐κB transcription factors where siRNA targeting p65 prevent senescence. Importantly, these IKK pathways are also relevant to ageing as knockout of *IKKA*, *IKKB* and *IKKE* avoid the activation of senescence. Altogether, these findings open a new potential line of investigation in the field of senescence by targeting the negative effects of the evSASP independent of particular EV contents.

## Graphical abstract

The evSASP mediates paracrine senescence via activation of IKK episilon, IKK alpha, IKK beta and the canonical NF‐kappa B pathway.

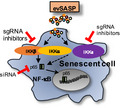

## 1 INTRODUCTION

The presence of senescent cells has been identified in many physiological and pathological conditions such as during embryonic development, cancer, ageing and several age‐related diseases (Di Micco et al., 2020; He & Sharpless, 2017; Munoz‐Espin & Serrano, 2014). Senescence is characterized by the activation of a complex and heterogeneous cellular phenotype presenting a stable cell cycle arrest. In spite of the lack of proliferative potential, senescent cells are highly metabolic and present elevated levels of transcription and translation. Furthermore, senescent cells have a very active secretome that has been denominated the senescence‐associated secretory phenotype (SASP). Thus, intercellular communication between senescent cells and their microenvironment is varied and can be mediated by soluble factors (Coppe et al., 2010). Other less characterized means of communication are extracellular vesicles, metabolites and ions, in addition to cell‐to‐cell contact, cell‐extracellular matrix interaction and cell fusion and cytoplasmic bridges (Fafian‐Labora & O'Loghlen, 2020). As these different means by which senescent cells communicate with their surroundings is context dependent, there is need for a better understanding of the molecular mechanisms and downstream pathways implicated.

In the last years, there has been an increasing interest in the role that extracellular vesicles (EV) play in the context of senescence, ageing and cancer (Fafian‐Labora & O'Loghlen, 2020). Many groups have identified different individual components enriched in the EV SASP (evSASP) such as microRNA (miR) (Jeon et al., 2019; Terlecki‐Zaniewicz et al., 2018), specific proteins (Basisty et al., 2020; Borghesan et al., 2019; Kavanagh et al., 2017; Takasugi et al., 2017) or nucleic acids (Takahashi et al., 2017). As with the soluble SASP (sSASP), the evSASP has detrimental effects on the microenvironment by inducing paracrine senescence in human primary fibroblasts (Borghesan et al., 2019), in proliferative chondrocytes in osteoarthritis (Jeon et al., 2019) or by promoting tumour progression (Takasugi et al., 2017). However, EV can also induce tissue repair by ameliorating senescence and ageing‐related tissue dysfunction (Fafian‐Labora et al., 2020; Yoshida et al., 2019).

In order to identify the molecular mechanisms and downstream pathways implicated in senescence mediated by the evSASP independent of single EV‐components, we performed a small molecule inhibitor screen and identified different signalling pathways including the NF‐κB pathway. NF‐κB proteins are implicated in a wide range of innate and adaptive immune responses that regulate cellular processes including senescence. NF‐κB is regulated by phosphorylation of one of its cytoplasmic inhibitors, IκB, mediated by IκB kinases (IKK), allowing NF‐κB nuclear translocation and transcriptional activation (Taniguchi & Karin, 2018). IKKα‐IKKβ heterodimer complexes regulate the DNA binding proteins implicated in the canonical NF‐κB pathway, including p50‐p65, while IKKα‐homodimers regulate p52‐RELB dimers implicated in the alternative NF‐κB pathway (Perkins, 2007; Taniguchi & Karin, 2018). On the other hand, IKKε can contribute to the regulation of NF‐κB but also type I interferon signalling (Shen & Hahn, 2011). Here, we find that pharmacological inhibition of IKKα, IKKβ and IKKε and knockout of their respective genes using CRISPR/Cas9 technology prevent senescence induced by the evSASP. We observe that the evSASP activates transcription factors mainly implicated in the canonical NF‐κB pathway and that this effect is dependent again on IKKα, IKKβ and IKKε. Furthermore, two independent RNAi targeting the NF‐κB canonical transcription factor p65 prevent the evSASP functionality. Altogether, we show that the evSASP activation of senescence is dependent on IKKα, IKKβ and IKKε and on canonical transcription factors binding to NF‐κB sequence specific oligonucleotides. Altogether, these findings are important as they open a new therapeutic line of research to block the unfavourable effects of the evSASP independent of individual evSASP content.

## 2 RESULTS

### 2.1 Small molecule inhibitor screen identifies different signalling pathways mediated by the evSASP

**FIGURE 1:**
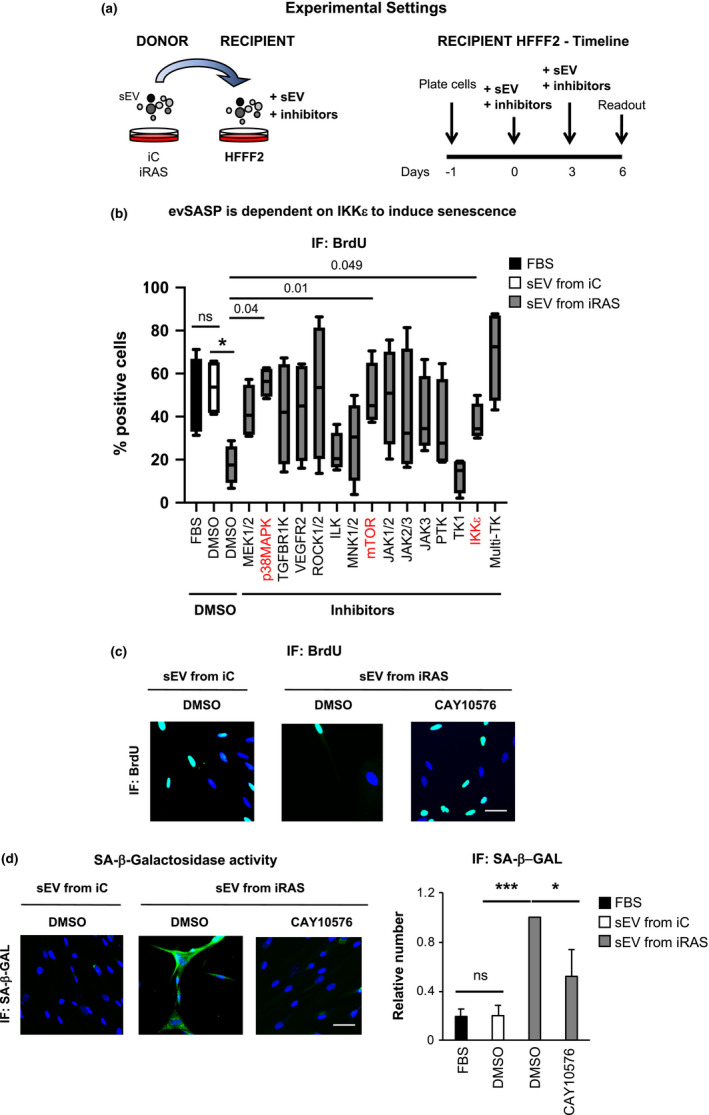
Small molecule inhibitor screen identifies different signalling pathways mediated by the evSASP (a) Details of the experimental settings to unveil novel signalling pathways mediated by the evSASP. Senescence was induced in iRAS donor cells by addition of 200 nM 4‐hydroxytamoxifen (+4OHT). HFFF2 recipient cells were treated with a panel of small molecule inhibitors in addition to sEV isolated from iC and iRAS cells. Drugs were maintained for 6 days and washed out. (b) Box and Whiskers plot representing the data obtained from the screen measuring proliferation by quantifying the percentage of HFFF2 that stain positive for BrdU. The graph indicates the targets of the small molecule inhibitors used: 40 µM PD98059 (targeting MEK1/2), 20 µM SB202190 (p38MAPK), 4 µM TGFB‐R1 (TGFBR1 kinase), 8 µM VEGFR2 (VEGFR2), 150 nM GSK429286A (ROCK1/2, Rho‐associated kinase), 50 nM CPD22 (ILK, integrin‐linked kinase), 1 µM CPG (MNK1/2), 100 nM TORIN2 (mTOR, mammalian target of rapamycin), 1µM RUXOLITINIB (JAK1/2 inhibitor), 40µM AG‐490 (JAK2/3 kinase), 45 µM JANEX1 (JAK3 kinase), 1 µM AG‐879 (protein Tyrosine Kinase), 2 µM IMATINIB (tyrosine kinase; TK1), 20 µM CAY10576 (IKKε, IκB kinase epsilon), 1.5 µM SUNITINIB (multi tyrosine kinase, multi‐TK). Data show the min to max Box and Whiskers plot of 4 independent experiments. Dunnett's multiple comparison test analysis was performed to determine statistical significance. p‐values are shown. (c) Representative IF pictures for BrdU staining in HFFF2 cells treated with or without 20 µM CAY10576 (IKKε inhibitor) and sEV from iC or iRAS. Scale bar: 50 µm. (d) Representative IF images (left panels) and quantification (right panels) of cells staining positive for senescence‐associated beta galactosidase activity (SA‐β‐Gal) in HFFF2 fibroblasts treated with 20 µM CAY10576 and sEV derived from iC or iRAS cells. Scale bar of IF pictures: 50 µm. All data represent the mean ± SEM of 4–5 independent experiments. Data were normalized to sEV from iRAS sample. One‐way ANOVA analysis was performed with multiple comparisons to the sEV iRAS control sample

Our laboratory has previously shown that small extracellular vesicles (sEV) in contrast to MV (microvesicles or larger vesicles >200 nm) are important mediators of intercellular communication between senescent and proliferative cells both *in vitro* and *in vivo* in the context of ageing and cancer (Borghesan et al., 2019; Fafian‐Labora & O'Loghlen, 2020; Fafian‐Labora et al., 2020). Although we have identified different proteins involved in both these contexts, the specific downstream signalling pathways are still unknown. To investigate this, we took advantage of healthy young human primary foreskin fibroblasts (HFFF2) expressing a chimeric form of the ligand‐binding oestrogen receptor (ER) fused to the active oncogene mutant H‐RAS^G12V^ as donor cells (named iRAS cells). The induction of senescence was achieved by adding 200 nM 4‐hydroxytamoxifen (4OHT) for 48 h followed by 72 h withdrawal in iRAS and control vector (termed iC) HFFF2 cells (Borghesan et al., 2019; Rapisarda et al., 2017). We isolated sEV from iC and iRAS cells by serial ultracentrifugation as performed previously (Borghesan et al., 2019; Fafian‐Labora et al., 2019; Fafian‐Labora et al., 2020; Thery et al., 2018) and treated recipient proliferating HFFF2 simultaneously with these sEV and a variety of pharmacological drugs inhibiting different signalling pathways for 6 days (Figure 1a). As we can see in Figure 1b, sEV isolated from iRAS treated with DMSO induced a cell cycle arrest measured by quantifying the percentage of cells incorporating BrdU. Media supplemented with EV‐depleted FBS was used as a control (FBS) and no differences were found between this condition and sEV from iC cells. However, HFFF2 treatment with inhibitors targeting p38MAPK, mTOR (mammalian target of rapamycin) and IKKε (IκB kinase epsilon) pathways prevented the proliferation arrest induced by sEV as part of the SASP (termed evSASP herein) (Figure 1b, c). IKKε is an essential regulator of innate immunity by regulating both the interferon and NF‐κB signalling pathway (Shen & Hahn, 2011). While we have previously identified the importance of the interferon pathway by the evSASP (Borghesan et al., 2019), the role that IKKε plays is unknown. To confirm the implication of the evSASP on the IKKε pathway, we determined additional markers of senescence and confirmed that IKKε inhibition using 20µM CAY10576 treatment for 6 days also reduced the high levels of senescence‐associated β‐galactosidase activity (SA‐β‐Gal), a common marker of senescence (Gorgoulis et al., 2019), as shown by IF pictures (left panel) and its corresponding quantification (graph) levels (Figure 1d). Next, we decided to determine the levels of another important hallmark of senescence, the cell cycle inhibitor p16^INK4A^. When looking at the number of cells staining positive for p16^INK4A^ by IF, we could also observe a decrease in these cells when treated with CAY10576 shown by IF pictures (above) and its relative quantification (graph below) (Figure S1a). To confirm that the results observed were not due to cytotoxicity by CAY10576, we determined whether CAY10576 was inducing apoptosis in the donor cells by staining with Annexin V and quantifying the number of cells staining positive by FACS (Figure S1b). Donor cells were used for this analysis to test pharmacological toxicity in proliferative *versus* senescent cells. Treatment of HFFF2 with 3 µM Cisplatin for 72 h was used as a positive control. Cytotoxicity was also determined in HFFF2 recipient cells simultaneously treated with sEV and CAY10576 by determining total cell number after treatment where no changes between DMSO and CAY10576 treatment was observed (Figure S1c). Altogether, these data suggest a role for IKKε in mediating senescence by the evSASP.

### 2.2 Pharmacological inhibition of IKKα and IKKβ prevents senescence mediated by the evSASP

**FIGURE 2:**
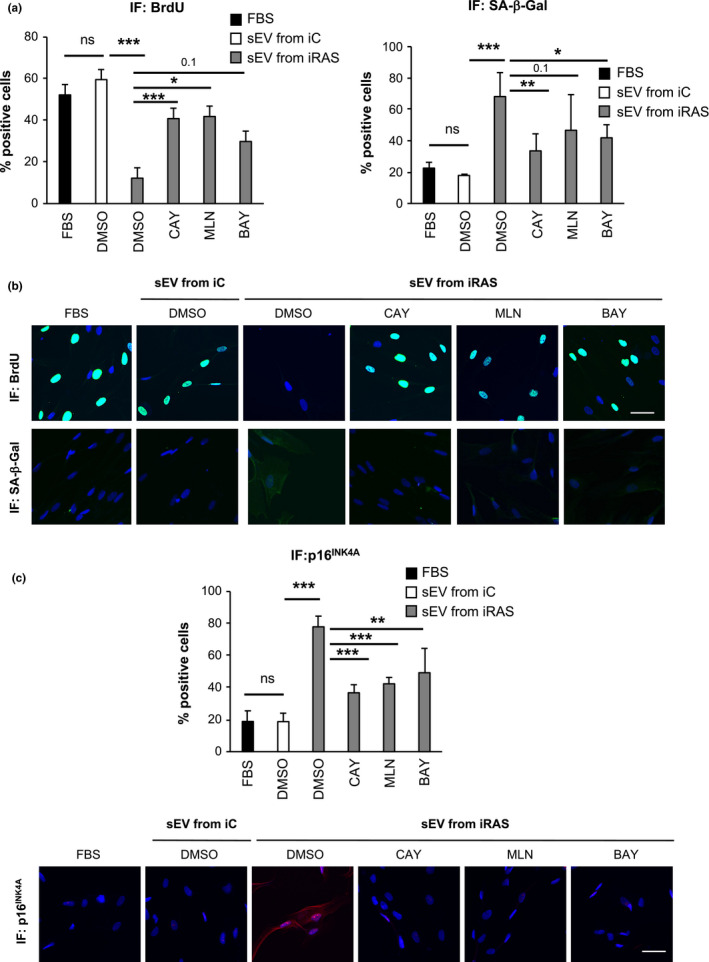
Pharmacological inhibition of IKKα and IKKβ prevents senescence mediated by the evSASP (a–c) HFFF2 recipient cells were treated with 20 µM CAY10576, 10 µM MLN120B or 10 µM BAY11‐7082 in addition to sEV isolated from iRAS cells for 6 days. The percentage of cells staining positive for (a, b) BrdU, SA‐β‐Gal and (c) p16^INK4A^ was determined. (a) Quantification of the percentage of cells staining positive for BrdU and SA‐β‐Gal and (b) representative pictures. (c) Quantification and pictures for p16^INK4A^ staining by IF. (a, c) Data show the mean ± SEM of 3 independent experiments. One‐way ANOVA with multiple comparisons with the DMSO/sEV from iRAS sample was performed. Scale bar: 50 µm

As IKKε contributes to regulate NF‐κB signalling pathway (Shen & Hahn, 2011), we next wanted to determine whether two key players, IKKα and IKKβ, were implicated in senescence mediated by the evSASP. For this, we used two commonly studied small molecule drugs that inhibit IKKα and IKKβ, respectively (10 µM BAY11‐7082 and 10 µM MLN120B for 6 days). Experiments were performed as in Figure 1a with CAY10576. Staining of HFFF2 cells for the apoptotic marker Annexin V and quantification by FACS show that none of these drugs induced cell toxicity in our system (Figure S2a). As expected, treatment with both BAY11‐7082 and MLN120B prevented the changes mediated by the evSASP (Figure 2). CAY10576 was used as a control in all experiments. BAY11‐7082 and MLN120B prevented the cell cycle arrest mediated by the evSASP measured by quantifying the percentage of cells incorporating BrdU by IF (Figure 2a, b). These drugs also induced a decrease in the percentage of cells staining positive for SA‐β‐Gal and p16^INK4A^ (Figure 2a–c). The percentage of cells staining positive for nuclear p65, a key component of the NF‐κB pathway, was also lower when HFFF2 cells were treated with BAY11‐7082 and MLN120B confirming the efficacy of the inhibitors in preventing nuclear p65 accumulation (Figure S2b, c). An additional p65 antibody was used to corroborate staining specificity (Figure S2c). Furthermore, to confirm that additional methods for sEV isolation would produce similar results and that sEV internalization was taking place, we isolated sEV from HFFF2 cells expressing an ectopic mCherry construct for human CD63 by size exclusion chromatography (SEC). We next pooled the sEV‐enriched fractions (fractions 3–7) (Figure S2d), performed and additional ultracentrifugation (UC) step and treated HFFF2 recipient cells as before. Both sEV and protein content were determined in 12 different fractions (Figure S2d). sEV internalization between the different conditions showed slight differences that were not statistically significant (Figure S2e, f).

We have previously shown that the evSASP induces DNA damage in recipient cells (Borghesan et al., 2019). Thus, to confirm that SEC‐isolated sEV activates senescence, we analysed the number of cells with DNA damage by staining with phospho‐γH2AX and observed that pharmacological inhibition of IKKα, IKKβ and IKKε prevented the DNA damage response induced by the evSASP with sEV isolated by SEC (Figure S2g).

### 2.3 Knockout of IKKα, IKKβ and IKKε using sgRNA prevents senescence mediated by the evSASP

**FIGURE 3:**
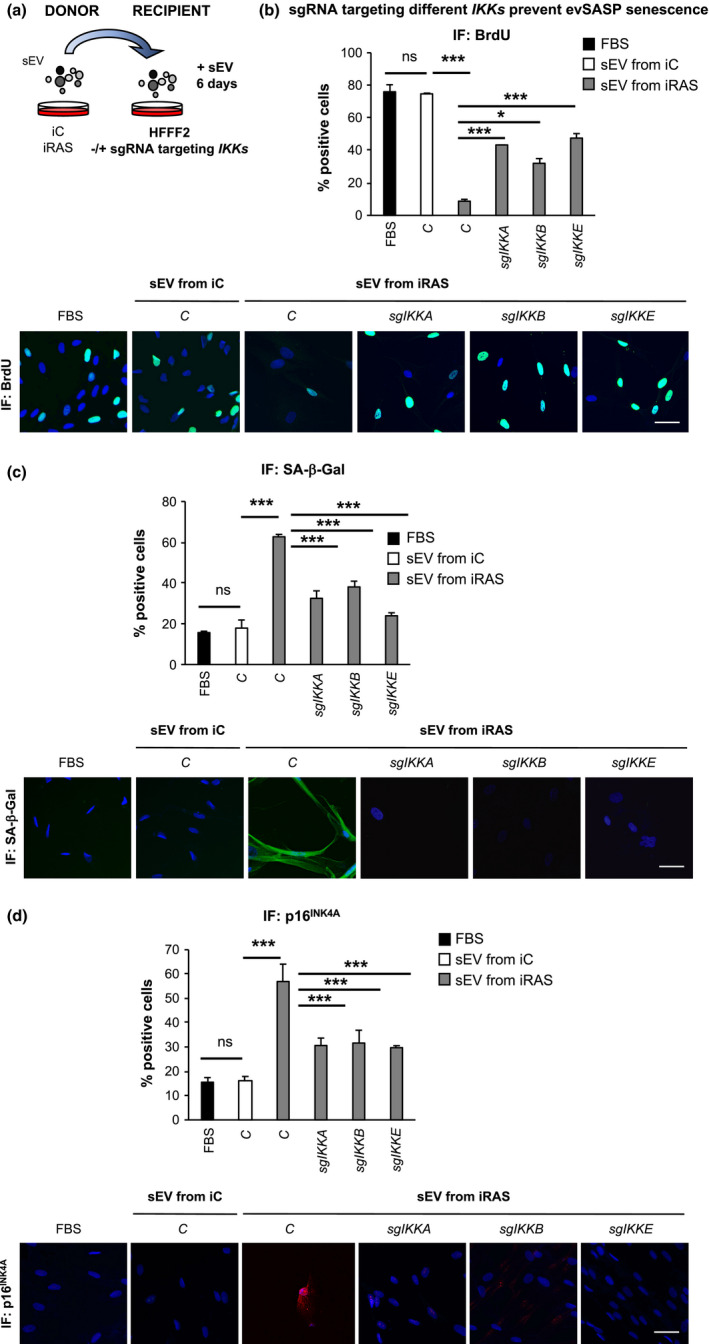
Knockout of IKKα, IKKβ and IKKε using sgRNA prevents senescence mediated by the evSASP (a) Representation showing the experimental settings where HFFF2 recipient cells expressing or not different sgRNA targeting IKKα (*sgIKKA*), IKKβ (*sgIKKB*), or IKKε (*sgIKKE*) were incubated with sEV from iC or iRAS for 6 days. (b–d) HFFF2 recipient cells transduced with *sgIKKA*, *sgIKKB* or *sgIKKE* were treated with sEV isolated from iC or iRAS cells for 6 days. The percentage of cells and representative pictures staining positive for (b) BrdU, (c) SA‐β‐Gal (d) and p16^INK4A^ is shown. Data show the mean ± SEM of 3 independent experiments. One‐way ANOVA with multiple comparisons to the C/sEV from iRAS sample was performed. Scale bar: 50 µm

To further determine the implication of IKKα, IKKβ and IKKε in evSASP‐mediated senescence, we proceeded to generate a single guide RNA (sgRNA) targeting *IKKA*, *IKKB* and *IKKE*, respectively, in a lentiviral vector containing a construct encoding for Cas9 and the sgRNA. We next transduced HFFF2 cells with 4 sgRNA per gene and after selection proceeded to treat these cells with sEV isolated from iC and iRAS cells for 6 days and determined different markers of senescence by IF (Figure 3a). As shown in Figure 3, knockdown of *IKKA*, *IKKB* and *IKKE* by sgRNA (*sgIKKA*, *sgIKKB* and *sgIKKE*) reversed the effects of the evSASP confirming the results obtained with the different inhibitors in Figure 2. In fact, *sgIKKA*, *sgIKKB* and *sgIKKE* partially prevented the cell cycle arrest induced by the evSASP (Figure 3b) and reduced the high levels of β‐Gal activity (Figure 3c). Furthermore, a decrease in the percentage of cells staining positive for p16^INK4A^ could also be observed upon the expression of *sgIKKA*, *sgIKKB* and *sgIKKE* (Figure 3d). The knockdown efficiency for all *IKKs* was confirmed by determining the mRNA levels by qPCR analysis using two different housekeeping genes (Figure S3a). The percentage of cells presenting p65 nuclear staining confirmed the disruption of NF‐κB pathway upon *IKKA*, *IKKB* and *IKKE* knockdown (Figure S3b).

### 2.4 Canonical NF‐κB pathway is activated by the evSASP

**FIGURE 4:**
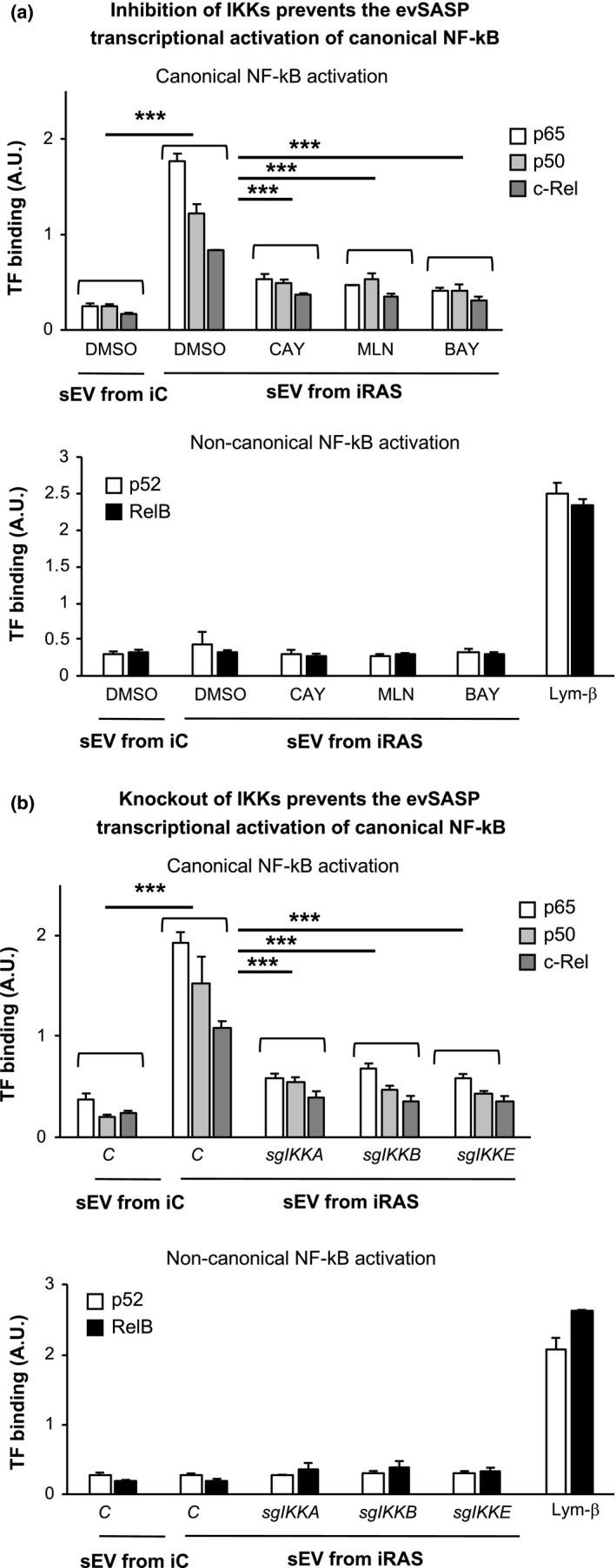
The canonical NF‐κB pathway is activated by the evSASP. Nuclear extracts were isolated from HFFF2 fibroblasts treated with (a) different IKKs inhibitors or (b) sgRNA targeting *IKK* and sEV from iC and iRAS simultaneously for 3 days. 5 ng/ml lymphotoxin‐β (Lym‐β) was used as a positive control for 8 h for the activation of the transcription factors p52 and RelB. Binding of canonical NF‐κB transcription factors, p65, p50 and c‐Rel, to an immobilized DNA fragment is shown upon treatment with sEV from iRAS and lost when treating with CAY10576, MLN120B and BAY11‐7082 IKKs inhibitors or in cells expressing sgRNA against *IKKs* constructs. No binding or changes upon the inhibitors treatments or sgRNA expression could be observed with the non‐canonical transcription factors: p52 and RelB. All data show the mean ± SEM of 3–6 independent experiments. One‐way ANOVA analysis was performed with multiple comparisons to the iRAS sEV control sample

To further confirm the implication of NF‐κB pathway in mediating evSASP senescence and to discern whether the canonical or non‐canonical pathways are implicated, we next performed a transcription factor high throughput assay to quantify binding of NF‐κB transcription factors to different oligonucleotides containing NF‐κB consensus binding sites. For this, we isolated nuclear extracts from HFFF2 treated with sEV from iC or iRAS cells and all 3 IKK inhibitors (20 µM CAY10576, 10 µM MLN120B or 10 µM BAY11‐7082) or expressing sgRNA targeting all 3 *IKKs* for 72 h. Binding of different transcription factors implicated in the canonical (p65, p50 and c‐Rel) or non‐canonical (p52 and RelB) activation of NF‐κB to a DNA sequence containing the NF‐κB consensus binding site was determined by ELISA. As shown in Figure 4, the evSASP induced the activation of the canonical NF‐κB pathway while no changes could be observed in the activation of factors regulating the non‐canonical pathways. Treatment of HFFF2 cells for 8 h with 5 ng/ml lymphotoxin‐β was used as a positive control for the activation of non‐canonical transcription factor‐binding activity. In agreement with our previous results, pharmacological inhibition of IKKα, IKKβ and IKKε (Figure 4a) or the genetic ablation of *IKKA*, *IKKB* and *IKKE* by sgRNA expression (Figure 4b) prevented the binding of all canonical transcription factors. Thus, these data further confirm that the canonical NF‐κB pathway is implicated in senescence mediated via the evSASP which is dependent on IKKα, IKKβ and IKKε although the contribution of each IKK is not determined.

### 2.5 siRNA targeting p65 prevents senescence mediated by the evSASP

**FIGURE 5:**
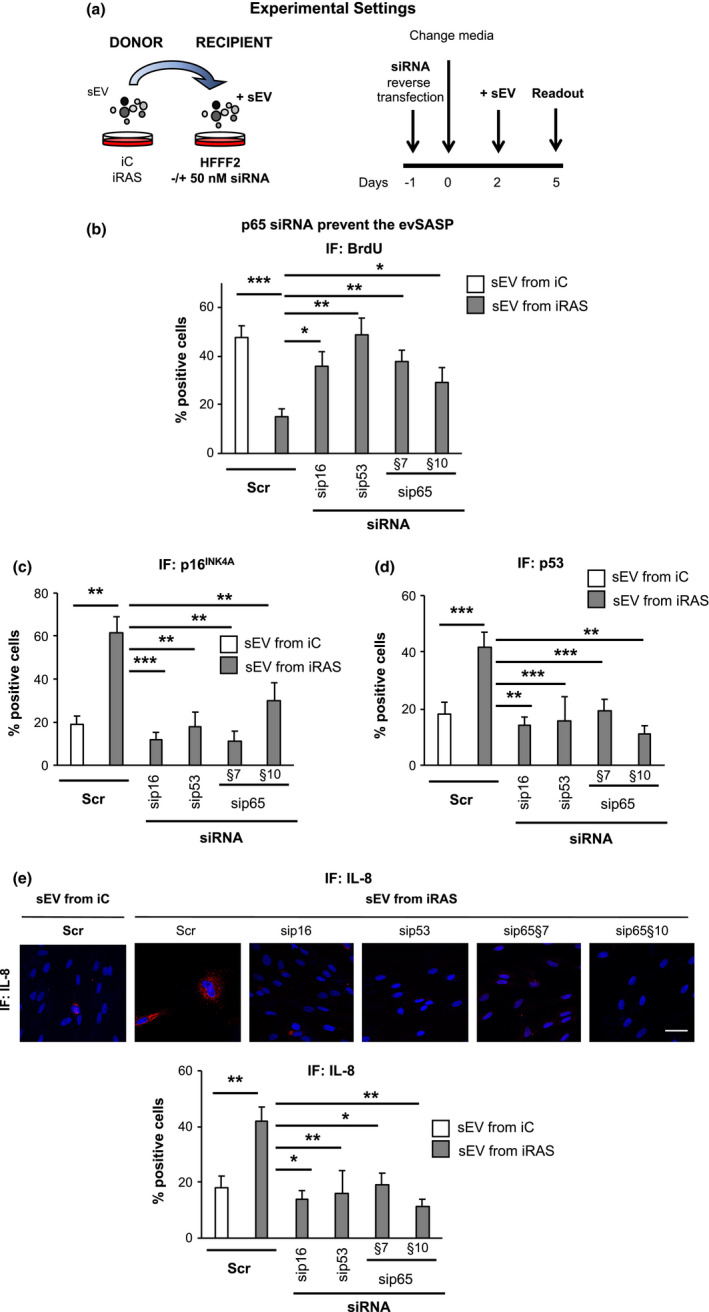
siRNA targeting p65 prevents senescence mediated by the evSASP. (a) Schematic representation for the experimental settings (left panel) and timings (right panel) of the experiments performed in this figure. Reverse transfection was performed with 50 nM of the indicated siRNA in the recipient cells. After washing the siRNA out, HFFF2 recipient cells were incubated with sEV isolated from either iC or iRAS for 72 h and 3 days later several markers of senescence were determined. (b) The use of two independent siRNA targeting p65 (sip65) ‐§7 and §10‐ prevents the cell cycle arrest characteristic of evSASP senescence. A scramble siRNA control (Scr) was used as a negative control while siRNAs targeting *CDKN2A* (encoding for p16^INK4A^) (sip16) or *TP53* (sip53) were used as positive controls. (c) The percentage of cells expressing p16^INK4A^ protein levels and (d) stabilizing p53 protein was quantified. (e) Representative IF images for IL‐8 expression levels in HFFF2 transfected with the indicated siRNAs and treated with sEV isolated from either iC or iRAS HFFF2 cells. Scale bar: 50 µm. Data show the mean ± SEM of 6 independent experiments. Statistical analysis was performed using One‐way ANOVA. All conditions were compared to sEV from iRAS control

To further confirm an implication of NF‐κB mediated by the evSASP, we took advantage of two independent siRNA targeting the canonical NF‐κB transcription factor, p65. A non‐targeting siRNA was used as a control (Scr). Furthermore, we used previously validated siRNA targeting two well characterized effectors of senescence: *TP53* (sip53) and *CDKN2A* (sip16) (Borghesan et al., 2019; Fafian‐Labora et al., 2019; Rapisarda et al., 2017) (Figure S4a). Here, 50 nM siRNA was reverse transfected simultaneously when plating the recipient HFFF2 cells. Media was changed 24 h later and sEV isolated from either iC or iRAS donor cells added 48 h later. The experiment was stopped 72 h after and several readout measures were taken (Figure 5a). First, we determined proliferation by quantifying BrdU incorporation. sEV derived from iRAS cells and transfected with Scr induced a cell cycle arrest by preventing the incorporation of BrdU, while sip53 and sip16 prevented the proliferation arrest induced by the evSASP (Figure 5b). Two independent siRNA targeting p65 (§7 and §10) also prevented the proliferation arrest mediated by evSASP (Figure 5b) concomitant with a decrease in the cell numbers staining positive for nuclear p65 (Figure S4b). Knockdown efficiency was validated at the RNA level (Figure S4c) and total cell number was determined to confirm that the siRNAs used were not inducing cell death (Figure S4d). To further confirm an implication for p65 by the evSASP we determined additional markers of senescence. The number of cells staining positive for p16^INK4A^ mediated by evSASP was also partially reduced by both p65 siRNA (§7 and §10) (Figure 5c) and sip53 and sip16. Furthermore, we could also observe that downregulation of p65 siRNA (§7 and §10) also prevented the stabilization of p53 mediated by the evSASP (Figure 5d). The reduction in the protein levels of p53 and p16^INK4A^ using their corresponding siRNA, sip53 and sip16, was confirmed by IF (Figure 5c, 5d). IL‐8 has been shown to induce senescence and its expression is regulated by p65 (Acosta et al., 2008). We therefore determined the levels of IL‐8 induced by the evSASP by protein (Figure 5e) and mRNA levels (Figure S4e) and the implication of p65. In fact, both siRNA targeting p65 siRNA (§7 and §10) reduced the number of cells staining positive for IL‐8 mediated by the evSASP at the protein level and prevented *IL8* mRNA upregulation (Figure 5e and Figure S4e). Altogether, these data suggest that the evSASP activates senescence through p65.

### 2.6 IKKs are important for evSASP senescence in human primary fibroblasts from young and old donors

**FIGURE 6:**
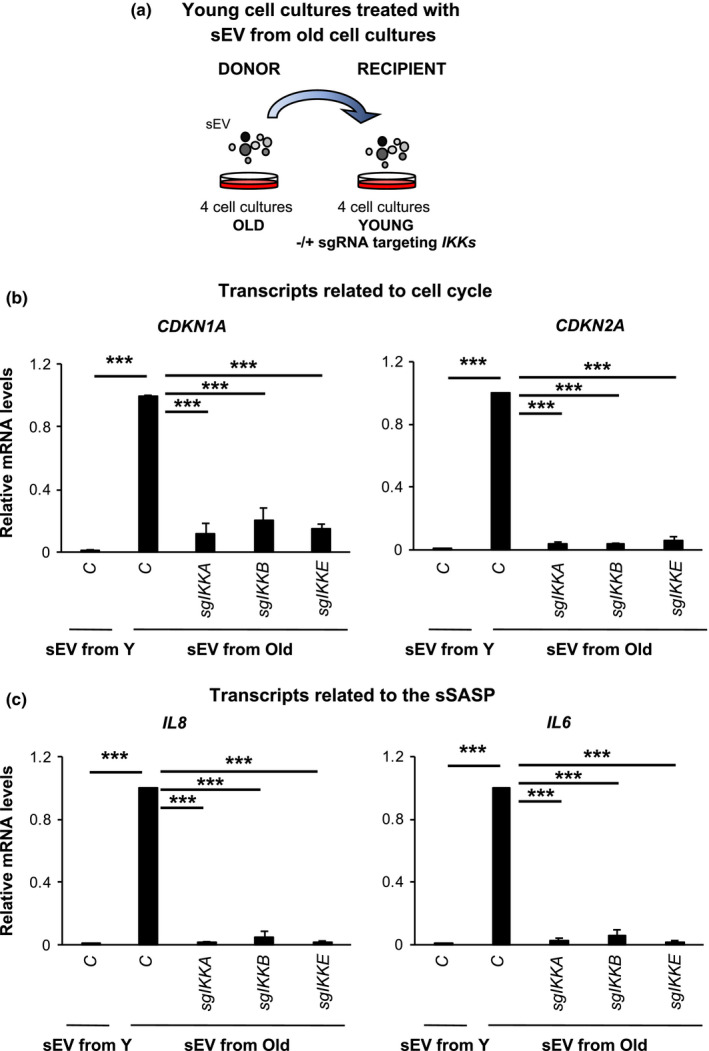
IKKs are important for evSASP senescence in human primary fibroblasts from young and old donors. (a) Schematic representation of the experimental settings where 4 human fibroblasts cell cultures isolated from young, Y, (~2 years) and old, O, (~70 years) donors were used. Pooled sEV isolated from four old cell cultures were used to treat four young cell cultures individually expressing and empty vector or sgRNAs targeting *IKK*s. (b, c) Relative mRNA levels for (b) the cell cycle regulators *CDKN1A* and *CDKN2A* or (c) components of the sSASP *IL8* and *IL6* were determined. All data represents the mean ± SEM of four individual cell cultures. One‐way ANOVA with Dunnett's multiple comparison analysis to control sEV old treated sample was performed. Data are normalized to the control sEV from the old donors sample

In order to determine the importance of NF‐κB and relevant IKKs in ageing, we took advantage of four human primary fibroblasts cell cultures isolated from young (~2 years) and four cultures from old (~70 years) donors. We have previously shown that fibroblasts from old donor express several markers of senescence (Fafian‐Labora et al., 2020; Rapisarda et al., 2017). All cells were used between passages 2–3 after arrival to prevent the onset of replicative senescence. Next, we pooled the conditioned media of all 4 old cell cultures, isolated their sEV and treated individually four young cell cultures expressing an empty vector or the different *sgIKKs* (Figure 6a). We next determined the expression levels of different markers of senescence by qPCR. We could observed that the treatment of all four young cell cultures for 6 days with sEV from old cell cultures induced an increase in the expression levels of different mRNA related to the cell cycle (Figure 6b) and the sSASP (Figure 6c). We could also observe that this induction of senescence was prevented by *sgIKKA*, *sgIKKB* and *sgIKKE*. The knockdown efficiency was confirmed by qPCR using two housekeeping genes (Figure S5). Thus, these data demonstrate that IKKα, IKKβ and IKKε play an important role in senescence mediated by the evSASP in human fibroblasts cultures from old and young individuals highlighting its relevance in ageing.

## 3 DISCUSSION

The SASP is one of the main means by which senescent cells communicate with their microenvironment in physiological and pathological conditions (He & Sharpless, 2017; Munoz‐Espin & Serrano, 2014). In fact, it is known that chronic inflammation or inflammaging causes tissue damage in ageing. One hypothesis for this is the accumulation of senescent cells and the effect of their associated SASP released to the microenvironment (Faget et al., 2019; Lee & Schmitt, 2019). While much research focuses on the discovery of novel drugs blocking the SASP (termed senomorphic drugs) (Di Micco et al., 2020), the off‐target effects of these drugs on organelle inter‐trafficking is understudied. During the maturation of the secretome in senescence, multiple organelles are likely to interact such as lysosomes, phagosomes and ER‐Golgi trafficking (Vizioli et al., 2020). In fact, dissecting the release of the soluble SASP (sSASP) from the evSASP proves difficult due to common pathways regulating inter‐organelle communication in senescence (Cavinato et al., 2020; Dou et al., 2017; Vizioli et al., 2020). Thus, an alternative approach to prevent the detrimental effects of the evSASP could be to prevent its effect on the microenvironment and neighbouring cells as described in our study, approach which has been successfully used in a model of liver regeneration (Bird et al., 2018).

Interestingly, the data presented here could open new therapeutic lines of research in the fields of senescence and ageing, i.e., by combining the use of senolytic drugs (that selectively kill senescent cells) with drugs preventing the downstream signalling of the SASP or even SASP components released upon the death of senescent cells. Senolytic drugs would not tackle the SASP already released prior to the elimination of senescent cells nor the SASP released upon the death of senescent cells. Therefore, blocking the downstream and paracrine signalling of the SASP after the use of the senolytics could reinforce the negative effect of senescence on ageing.

It is interesting that our data show dependence of different pathways to activate senescence by the evSASP. This could be considered as a limitation of our study, as theoretically several pathways would need to be inhibited to prevent the detrimental effect of the evSASP. However, the fact that most pathways identified in this study lead to a particular common pathway, NF‐κB, is an advantage (Mowla et al., 2013). Inhibition of NF‐κB would not only act as a senomorphic drug but would also prevent evSASP senescence. NF‐κB is constitutively activated during senescence and is considered a key regulator of the sSASP, where inhibition or knockout of several components that modulate NF‐κB signalling, for example p65, have proven to dampen the sSASP (Di Micco et al., 2020; Fafian‐Labora & O'Loghlen, 2020). In contrast, the non‐canonical factors NFKB2 and RelB have been shown to suppress senescence via the epigenetic regulator EZH2 (Iannetti et al., 2014). Although activation of the sSASP has been mainly attributed to a persistent and unrepairable DNA damage response, other stressors such as p38MAPK and cytoplasmic chromatin fragments (CCFs) also activate the sSASP through NF‐κB (Fafian‐Labora & O'Loghlen, 2020). However, the interconnection between the different IKK in senescence are understudied. Several RNA‐seq studies see an increase in *RELA*, *RELB* and *NFKB2* transcripts in senescence (Acosta et al., 2013; Borghesan et al., 2019; Herranz et al., 2015; Storer et al., 2013), but few reports show an increase in *IKK* mRNA as in this study. However, our results show an increase in *IKK* mRNA using different housekeeping genes with no significant differences between their expressions in the various experimental conditions, suggesting a transcriptional regulation upon treatment with the evSASP. Interestingly, a recent study has shown that during the initial activation of senescence, IKKβ is activated by ATM while in the later stages the sSASP is expressed independently of IKKα/β and proteasome degradation of IκB in spite of depending on the NF‐κB protein p65 (Kolesnichenko et al., 2021). Altogether, this suggests that the activation of NF‐κB is far more complicated than initially thought in the context of senescence and both the sSASP and evSASP.

The fact that the evSASP activates the IKKα, IKKβ and IKKε pathways could be a reflection of different things. The evSASP could be activating a common upstream regulator of the NF‐κB pathway such as IKKβ, resulting in the nuclear translocation of p65, with a direct or indirect contribution of IKKα and IKKε. In fact, when manipulating a single sEV protein component such as the interferon protein IFITM3, theoretically targeting a particular evSASP subpopulation, we only see partial amelioration of the senescence phenotype mediated by the evSASP (Borghesan et al., 2019) indicating that other factors are essential or that other sEV populations can mediate evSASP senescence. The other alternative is that we are isolating a heterogeneous sEV population (Tkach & Thery, 2016) where one sEV subpopulation could be targeting IKKβ while another sEV subpopulation within the same sample could be targeting IKKα/IKKε. Interestingly, in all cases, we see induction of senescence and activation of p65. This is not uncommon as EV has been shown to activate NF‐κB via TLR signalling in other contexts resulting in the induction of pro‐inflammatory cytokines by human monocytic cells (Bretz et al., 2013) or during parasite infection (Toda et al., 2020).

Importantly, we see that the evSASP employed using the iRAS model of senescence represents what happens using an *in vitro* model of ageing. Thus, the downstream signalling pathways involving IKKα, IKKβ and IKKε observed by four different primary fibroblasts isolated from human donors aged between 67 and 80 years old on four different fibroblasts isolated from donors aged 1–3 years old are conserved between both models. Altogether, these results highlight the importance of the IKKα, IKKβ and IKKε signalling pathways in activating senescence by the evSASP in a model of oncogene senescence and ageing. Furthermore, these results could open new lines of research for therapeutically targeting senescence in ageing and age‐related diseases.

## 4 EXPERIMENTAL PROCEDURES

### 4.1 Cell cultures

HFFF2 human foreskin primary male fibroblasts were obtained from the Culture Collections (Public Health England). Young and old primary human fibroblasts cell cultures were obtained from the Coriell Cell Repository with the following codes: Young: GM05399 (1 year old; male), GM00969 (2 years old; female), GM05565 (3 years old; male), GM05758 (1 year old; male); Old: AG16086 (67 years old; female), AG06240 (80 years old; male), AG13152 (80 years old; male), AG13222 (81 years old; male). Details on cell passage are described as following:

- GM05399‐Young; Arrived passage 4 ‐ Used at passage 7 in the experiment
- GM00969‐ Young; Arrived passage 14 ‐ Used at passage 16 in the experiment
- GM05565‐Young; Arrived passage 7‐ Used at passage 9 in the experiment
- GM05758‐ Young; Arrived passage 4 ‐ Used at passage 7 in the experiment
- AG16086‐ Old; Arrived passage 5 ‐ Used at passage 7 in the experiment
- AG06240‐Old; Arrived passage 15 ‐ Used at passage 17 in the experiment
- AG13152‐ Old; Arrived passage 5 ‐ Used at passage 7 in the experiment
- AG13222‐ Old; Arrived passage 5 ‐ Used at passage 7 in the experiment

Cells were maintained in high‐glucose, pyruvate, Dulbecco's modified Eagle's medium with 10% foetal bovine serum and 1% antibiotic‐antimycotic (A/A) solution in a 37°C incubator with 5% CO_2_. iC and iRAS HFFF2 cultures were plated in a 100 mm dish. After 24 h, the cells (iC and iRAS) were treated with 200 nM 40HT for 48 h in DMEM supplemented with 10% (v/v) FBS and 1% A/A. Thereafter, the cells were washed and maintained with 0.5% (v/v) in EV‐depleted FBS and 1% (v/v) A/A for 3 days to collect the medium to isolate sEV.

### 4.2 Small molecule inhibitor screen

HFFF2 recipient cells were treated with several inhibitors at the below concentrations simultaneously with sEV in medium supplemented with 10% (v/v) EV‐depleted FBS and 1% (v/v) A/A for 72 h. Treatment with inhibitors and sEV was repeated 72 h later and readout was determined after 72 h.

Small molecule inhibitor concentrations used for the screen: 40 µM PD98059 (targeting MEK1/2; ThermoFisher), 20 µM SB202190 (p38MAPK; Santa Cruz Biotech), 4µ M TGFB‐R1 (TGFBR1 kinase; Calbiochem), 8 µM VEGFR2 (VEGFR2; Calbiochem), 150 nM GSK429286A (ROCK1/2, Rho‐associated kinase; Abcam), 50 nM CPD22 (ILK, integrin‐linked kinase; Calbiochem), 1 µM CPG (MNK1/2; Calbiochem), 100 nM TORIN2 (mTOR, mammalian target of rapamycin; CAYMAN Chemical), 1µM RUXOLITINIB (JAK1/2 inhibitor; CAYMAN Chemical), 40 µM AG‐490 (JAK2/3 kinase; CAYMAN Chemical), 45 µM JANEX1 (JAK3 kinase; CAYMAN Chemical), 1 µM AG‐879 (protein Tyrosine Kinase; CAYMAN Chemical), 2 µM IMATINIB (tyrosine kinase; TK1; CAYMAN Chemical), 20 µM CAY10576 (IKKε, IκB kinase episilon; CAYMAN Chemical), 1.5 µM SUNITINIB (multi tyrosine kinase, multi‐TK; CAYMAN Chemical). Additional small molecular inhibitors used: 10 µM MLN120B (IKKβ, IκB kinase beta; MedChemExpress) and 10 µM BAY11‐7082 (IKKα/β, IκB kinase alpha/beta; Sigma‐Aldrich). Other pharmacological products: lymphotoxin‐β (Sigma Aldrich‐T7799) and Cisplatin (PHR1624; Sigma‐Aldrich).

### 4.3 siRNA reverse transfection

HFFF2 cells were reverse transfected with 50 nM siRNAs on a well of a 96‐well plate. After 24 h, the medium was changed. Two days later, the cells were washed and incubated with the sEV in medium supplemented with 10% (v/v) EV‐depleted FBS and 1% (v/v) A/A for 72 h. siRNAs used in this study are below:

**Table:** 

siRNA: p16	SI02623747	QIAGEN
siRNA: p53	SI02664403	QIAGEN
siRNA: p65§7	SI02663094	QIAGEN
siRNA: p65§10	SI04437062	QIAGEN

### 4.4 Retroviral and lentiviral infections

The generation of stable retroviral and lentiviral expression was carried out following previous studies (Acosta et al., 2008; Borghesan et al., 2019; Rapisarda et al., 2017). Briefly, retroviral particles were generated by transfecting ER‐HRAS^G12V^ plasmid and retroviral helper plasmids (vsvg and gag‐pol) with Polyethylenimine (PEI) in HEK293T packaging cells for 48h. Recombinant lentiviral particles were generated using the second‐generation packaging vectors psPAX2 and pMD2.G using PEI in HEK293T. The supernatant was then filtered with 0.45µm filters (Starlab) and applied to HFFF2 cells in the presence of 4 µg/ml polybrene (hexadimethrine bromide; Sigma‐Aldrich) following threee rounds of infection. Cells were subsequently selected with the appropriate antibiotic resistance either 0.5 μg/ml puromycin or 300 μg/ml neomycin (Invitrogen).

For infections with the sgRNA, a pool for the 4 sgRNA (5 μg per single sgRNA) targeting a single *IKK* was generated by transfecting equal amounts of DNA and the packaging vectors psPAX2 and pMD2.G. Infection was performed as described earlier for lentivirus.

### 4.5 CRISPR sgRNA generation

We used the online guide design tool (http://crispr.mit.edu) to identify ideal sgRNA sequences. The highest scoring sgRNA guides were selected. Primers for the below sequences were ordered and the complementary primers annealed at 37°C for 30min, followed by at 95°C for 5 min and then ramped down to 25°C at 5°C degrees per min. The annealed synthetic sgRNA oligonucleotides were cloned into pLentiCRISPR v2 vector (Addgene #52961) at BsmBI restriction sites. The sgRNA sequences are as follows:

*sgIKKA*

1. ACAGACGTTCCCGAAGCCGC
2. GCATGAGAAGATTAAGAAGA
3. AATTGGGACCCTCAGCAGAG
4. GCAATGGAATACTGTTCTGG

*sgIKKB*

1. GCCGTCTGAAATACACTGAG
2. AGTGCGGCAGAAGAGTGAGG
3. GCCGAAGCTCCAGTAGTCGA
4. ACTGCACGGGCTGCCAGTTG

*sgIKKE*

1. GAGGTACTCCTGGTGTCGGG
2. TGCATCGCGACATCAAGCCG
3. ACAGCACATCGCCCACACGA
4. TGGCCGGCATGAACCACCTG

### 4.6 sEV isolation and treatment

To isolate the sEV fraction, the protocol of differential ultracentrifugation (Thery et al., 2018) was modified and adapted (Borghesan et al., 2019; Fafian‐Labora et al., 2020). Briefly, 1 × 10^6^ early passage donor cells were plated in a 10 cm dish (10 ml media) for each individual experiment in high glucose Dulbecco´s modified Eagle´s medium with 10% (v/v) EV‐depleted foetal bovine serum (FBS) and 1% (v/v) antibiotic‐antimycotic solution (A/A). 72 h prior to sEV collection media was switched to 0.5% EV‐depleted FBS except for the young and old human primary fibroblasts cell cultures that were kept ii 10% EV‐depleted FBS. The Conditioned Media was then collected by pipetting from the 10cm dish in 50 mL falcon tubes, centrifuged at low speed (2,000 *g* for 20 min; k‐factor of the rotor is 41056) to eliminate dead cells and cellular debris. This was followed by a 10,000 *g* centrifugation step for 1 h. The supernatant was then filtered through a 0.22 µm filter and we further proceeded to the final 100,000 *g* centrifugation step for 1 h and 20 min. The 100,000 *g* pellet (sEV) was washed once in 15 ml of PBS and resuspended in 10% EV‐depleted FBS media for the functional cell culture experiments. In summary, we resuspend the final 100,000 *g* pellet in 840 µl EV‐depleted media and treated 12 wells of a 96‐well plate (plated with 1,500 recipient cells each) with 70 µl of the sEV resuspension. Thus, with 10 ml of conditioned media from one 10cm dish (1x10^6^ cells) from either iC or iRAS cells we treated 12 wells of a 96 well plate. Media with EV‐depleted serum (FBS10%) was used as negative control in most experiments. Although iRAS cells release more sEV than iC cells, we previously validated that the induction of paracrine senescence was due to sEV content rather than sEV number (Borghesan et al., 2019). A Sorvall 100SE Ultra Centrifuge, with a Beckmann Fixed Angle T865 rotor was used for sEV isolations. The *k*‐factor of the rotor is 2,08.

### 4.7 Size exclusion chromatography sEV isolation

For size exclusion chromatography (SEC), we used qEV columns (Izon Science). Twelve eluted fractions were collected in sequential fractions of 1ml according to the manufacturer's instructions. The particle and protein concentration of each fraction was then measured by NTA and MicroBCA. The fractions enriched in particles and lacking protein contaminants were pooled, centrifuged at 100,000 *g* for 1 h 20 min and used for functional assays.

### 4.8 Transcription factor binding assay NF‐κB

The protocol was performed following the manufacturers instructions' for the NF‐κB Transcription Assay Kit (Colorimetric) (ab207216, Abcam). Firstly, the nuclear extraction was achieved using the nuclear lysis buffer. Nuclear extracts were incubated with oligonucleotides containing NF‐κB consensus binding sites for 1 h at room temperature with mild agitation. After washing with 1 × wash buffer, they were incubated with primary antibodies implicated in the canonical and non‐canonical NF‐κB pathway (1:1000) for 1 h at room temperature. After washing again with 1 × wash buffer, the samples were incubated with HRP‐conjugated secondary antibody (1:1000) for 1 h at room temperature and washed. The developing solution was added to the samples until a colorimetric reaction could be observed after which the Stop Solution was added. The absorbance was measured at OD 450 nm using Synergy HT Multi‐Mode Microplate Reader.

### 4.9 FACS for Annexin V staining

Apoptosis assay was quantified using the Annexin V, Alexa Fluor™ 488 conjugate (ThermoFisher Scientific, Cat. No. A23204). Briefly, cell medium and cells were collected in a FACS tubes. The cells were incubated with Annexin V‐488 (25 µg/ml) prepared in 1X Annexin Binding Buffer (ThermoFisher, Cat. No. PNN1001) for 10 min in the darkness at room temperature. Thereafter 1 µg/ml PI staining solution was added and FACS was carried out using the NovoExpress Software (Acea, Bio). The flow cytometer used for Annexin V detection is the ACEA Novocyte (Agilent).

### 4.10 qPCR analysis

RNA was isolated using TRIzol Reagent (Thermo Fisher) from the cells washed with PBS. cDNA was generated using the High‐Capacity cDNA Reverse Transcription Kit (ThermoFisher) according the manufacturer´s instructions. qPCR was performed using SYBR Green PCR Master Mix (Applied Biosystems) on a 7500 Fast System RealTime PCR cycler (Applied Biosystems). Primers sequences used in this study are:

- *RPS14*: For CTGCGAGTGCTGTCAGAGG; Rev TCACCGCCCTACACATCAAACT
- *ACTB*: For GCCCTGAGGCACTCTTCCA; Rev CGGATGTCCACGTCACACTTC
- *IL8*: For GAGTGGACCACACTGCGCCA; Rev TCCACAACCCTCTGCACCCAGT
- *IL6*: For CCAGGAGCCCAGCTATGAAC; Rev CCCAGGGAGAAGGCAACTG
- *CXCL1*: For GAAAGCTTGCCTCAATCCTG; Rev CACCAGTGAG CTTCCTCCTC
- *IKKA*: For AATGTGTTTTTCCCCCATGA; Rev AGGCAAATGTGTCGTGATGA
- *IKKB*: For AACCAGCATCCAGATTGACC; Rev CTCAGGTCGTCCAGCGTTC
- *IKKE*: For CTGTTCTGTGGCTGCCTGTA; Rev GAGAAGCAGGTCCTTTCGTG
- *CDKN2A*: For CGGTCGGAGGCCGATCCAG; Rev GCGCCGTGGAGCAGCAGCAGCT
- *CDKN1A*: For CCTGTCACTGTCTTGTACCCT; Rev GCGTTTGGAGTGGTAGAAATC
- *TP53*: For CCGCAGTCAGATCCTAGCG; Rev AATCATCCATTGCTTGGGACG
- *RELA*: For TTCCCGATCTGAGTCCAGGT; Rev GCTTGTCTCGGGTTTCTGGA

The relative expression was calculated using the ΔΔCt methods employing the Ct values generated with the 7500software version 2.0.6 (Applied Biosystems). The data were normalized to the housekeeping gene, *RPS14* or *ACTB* whenever specified.

### 4.11 Nanoparticle tracking analysis

The NanoSight LM10 (Malvern Instruments) was calibrated using Silica Microspheres beads (Polyscience). sEV were diluted in PBS (Sigma‐Aldrich) in order to obtain a particle number between 10^8^–10^9^ particles. Three measurements of 60 s were taken per each sample and the mean value was used to determine particle number. The movement of each particle in the field of view was measured to generate the average displacement of each particle per unit time, which was calculated using the Nanoparticle Tracking Analysis (NTA) 3.0 software (Malvern Instruments).

### 4.12 SA‐β‐Galactosidase activity

The cells were incubated with 33 µM of the β‐galactosidase substrate C12FDG (Fluorescein di‐β‐D‐galactopyranose) (F2756 from Sigma‐Aldrich) in medium supplemented with 0.5% (v/v) EV‐depleted FBS for 8 h at 37wC. After the incubation, the cells were washed with PBS and fixed with 4% (v/v) paraformaldehyde (Sigma‐Aldrich) for 15 min at room temperature.

### 4.13 Immunofluorescence staining

To determine BrdU incorporation, 50 µM of the thymidine analogue BrdU was added to the live cell cultures approximately 16 h before the end of the experiment. For all immunofluorescence, staining cells were washed with PBS and fixed in 4% (v/v) paraformaldehyde (Sigma‐Aldrich) for 15 min at room temperature. After, the cells were washed in PBS twice and permeabilized with 0.2% (v/v) Triton ×‐100 (Sigma‐Aldrich). Then, the cells were blocked with 1% (m/v) BSA and 0.2% (m/v) gelatin fish (All from Sigma‐Aldrich). After, the cells were incubated with the concentration of primary antibody detailed below. For BrdU detection, the cells were incubated with 0.5 U/µl DNaseI and 3 mM MgCl_2_ (All from Sigma‐Aldrich) overnight at 4°C. The next day, the preparations were washed with PBS twice and incubated with DAPI and secondary antibody at a concentration of 1:500. Finally, the cells were washed with PBS twice and the IF preparation were maintained with PBS and stored at 4°C until the acquisition of pictures using IN Cell 2200 automatized microscope (GE). Antibody concentrations used: p16^INK4A^ (Abcam, ab108349; 1:500), BrdU (Abcam, ab6326; 1:500), p65 (Abcam, AB_10859369; 1:500 and F6, Santa Cruz_sc8008; 1:500), IL‐8 (R&D, MAB208; 1:500), p53 (Santa Cruz Biotech, sc‐126; 1:200).

### 4.14 High content analysis immunofluorescence

The images of immunofluorescence were acquired using the automated high throughput fluorescent microscope IN Cell Analyzer 2000 (GE Healthcare) with a Å~20 objective. The fluorophores in wavelength settings were used to generate the IF images (‘DAPI’ for DAPI, ‘FITC’ for AlexaFluor 488 FITC, and ‘Cy5’ for AlexaFluor647). Ten fields per well were acquired to include a minimum of 100 cells per sample well.

High content analysis (HCA) of the images were processed using the IN Cell Investigator v.2.7.3 software as described previously (Borghesan et al., 2019). DAPI was used as a nuclear mask and a top‐hat method allowed the segmentation of cells. To detect cytoplasmic staining in cultured cells, a collar of 7–9 nm around DAPI was applied. Nuclear staining in the reference wavelength, that is, all the other wavelengths apart from DAPI, was quantified as an average of pixel intensity (grey scale) within the specified nuclear area. The Cytoplasmic IF was quantified as a coefficient of variance of the pixel intensities within the collar area in the reference wavelength. In samples of cultured cells, a threshold for positive cells was assigned above the average intensity of unstained or negative control samples.

### 4.15 Statistics

Results are represented as the mean ± S.E.M except where specified. Statistical analysis are specified in the figure legend. Samples were compared to the senescent sample unless specified otherwise. Significances are considered as following: *p < 0.05; **p < 0.01; ***p < 0.001.

## CONFLICT OF INTEREST

AO’s lab is funded by Starklabs in a project unrelated to these data. AO is part of Starklabs Scientific Advisory Board.

## AUTHOR CONTRIBUTIONS

J.A.F.‐L. performed all the experiments and data analyses. J.A.F.‐L. and A.O. wrote the manuscript. A.O. conceived and designed the study.

## Supporting information

Fig S1‐S5

## Data Availability

The data that support the findings of this study are available in the supplementary material of this article.
