# Supplementary material for: NF‐κB/IKK activation by small extracellular vesicles within the SASP
Source: Aging Cell. 2021 Jun 29;20(7):e13426. doi: 10.1111/acel.13426 (PMC8282244; doi:10.1111/acel.13426)
Supplement: Supplementary file 1 — Fig S1‐S5 [file ACEL-20-e13426-s001.pdf]

(a)

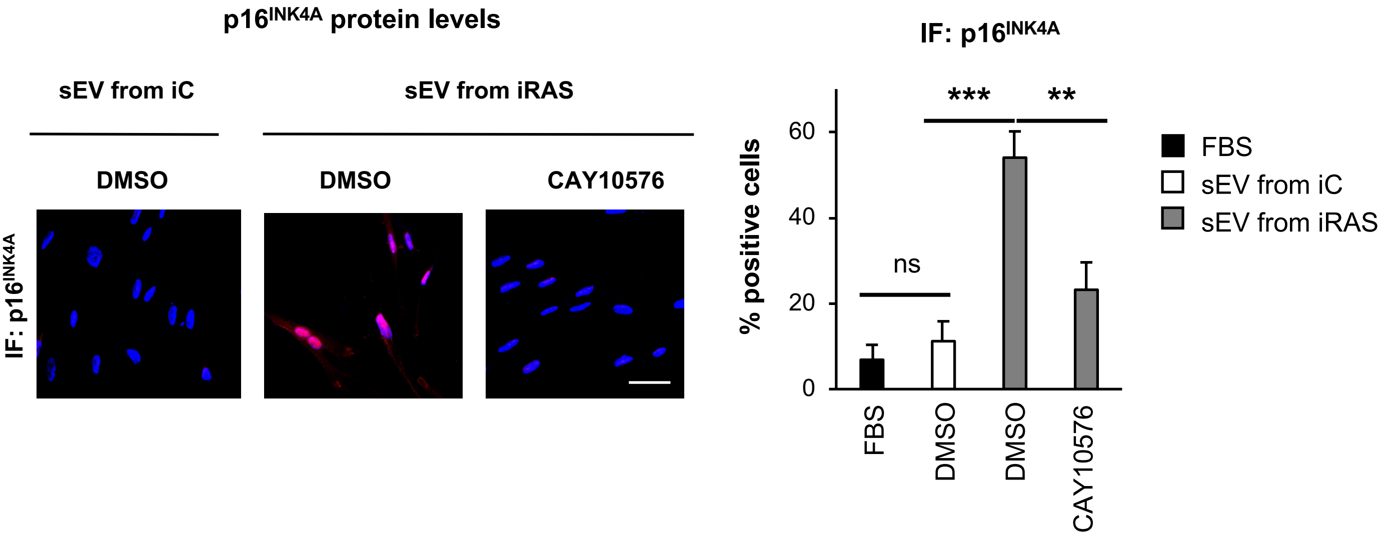
p16INK4A protein levels
sEV from iC
sEV from iRAS
DMSO
DMSO
CAY10576
IF: p16INK4A
IF: p16INK4A
% positive cells
60
40
20
0
ns
***
**
FBS
DMSO
DMSO
CAY10576
FBS
sEV from iC
sEV from iRAS

(b)

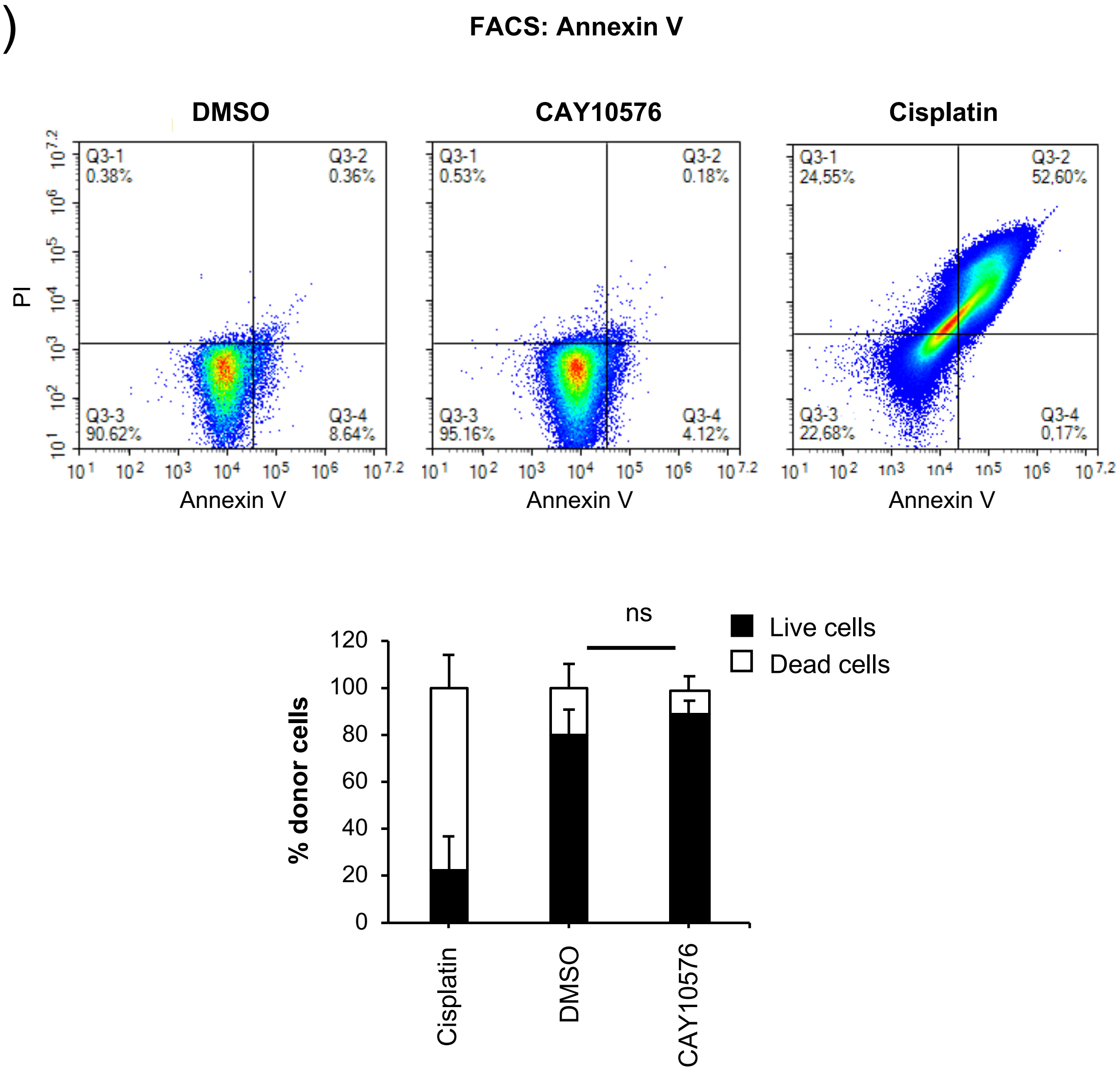
FACS: Annexin V
DMSO
CAY10576
Cisplatin
PI
Q3-1 0.38%
Q3-2 0.36%
Q3-3 90.62%
Q3-4 8.64%
Q3-1 0.53%
Q3-2 0.18%
Q3-3 95.16%
Q3-4 4.12%
Q3-1 24,55%
Q3-2 52,60%
Q3-3 22,68%
Q3-4 0,17%
Annexin V
Annexin V
Annexin V
% donor cells
120
100
80
60
40
20
0
ns
Live cells
Dead cells
Cisplatin
DMSO
CAY10576

(c)

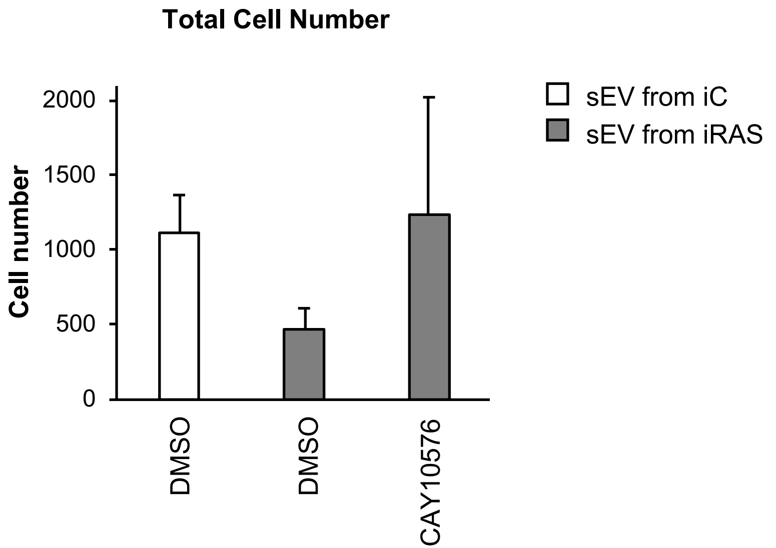
Total Cell Number
Cell number
2000
1500
1000
500
0
sEV from iC
sEV from iRAS
DMSO
DMSO
CAY10576

Fafián-Labora *et al.* **Figure S2**

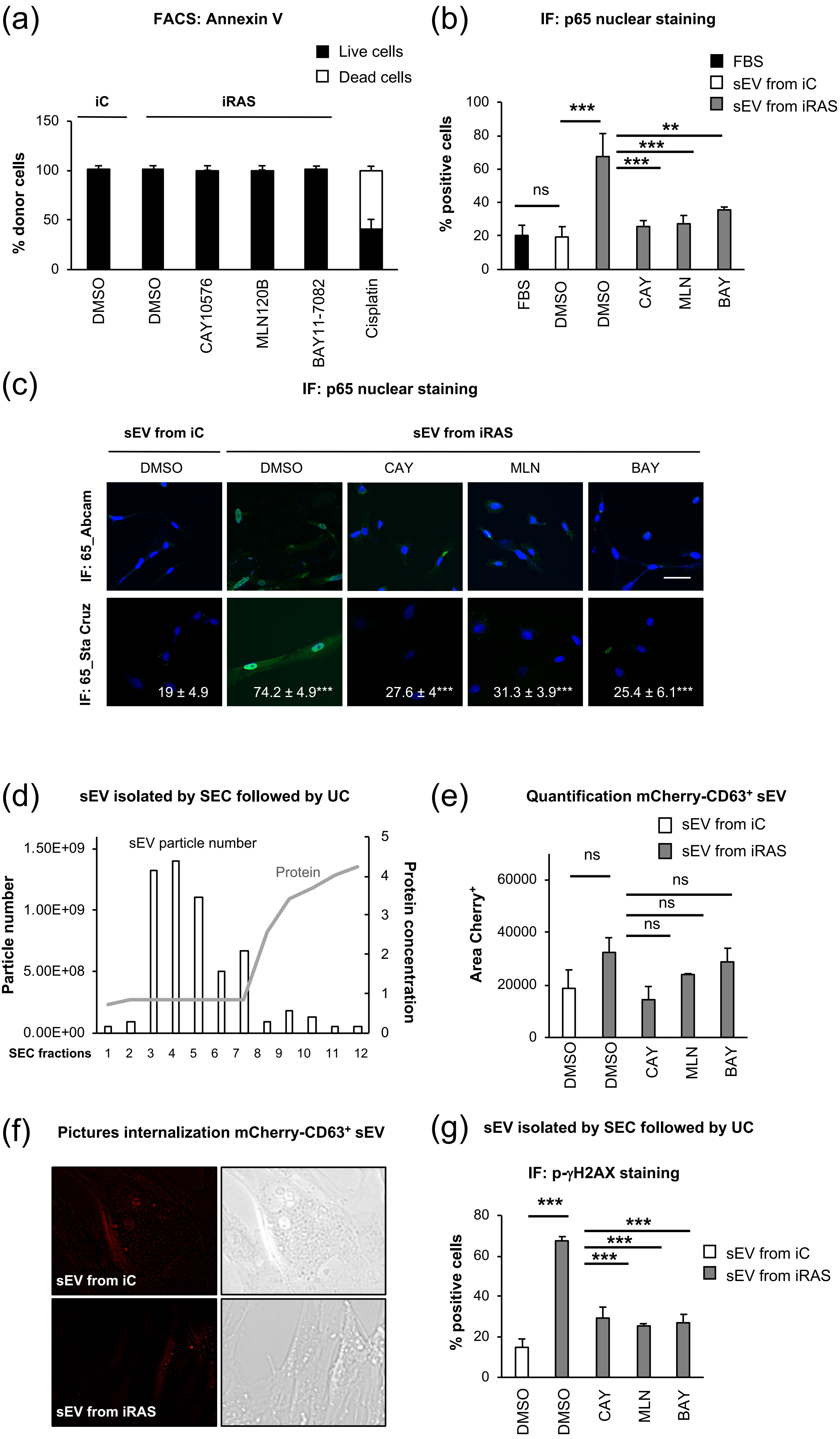

(a)

sgRNA knockdown efficiency

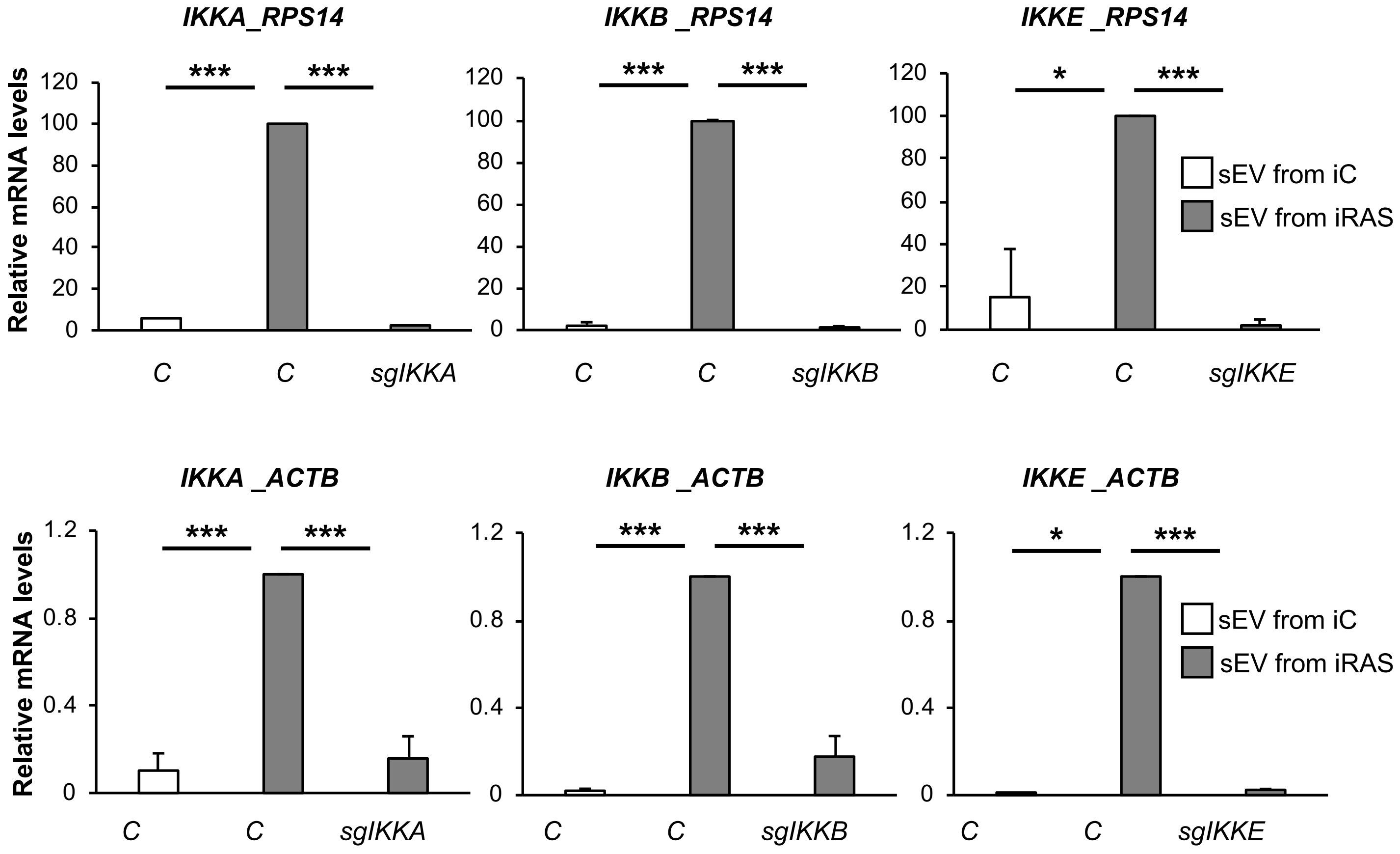

(b)

IF: p65 nuclear staining

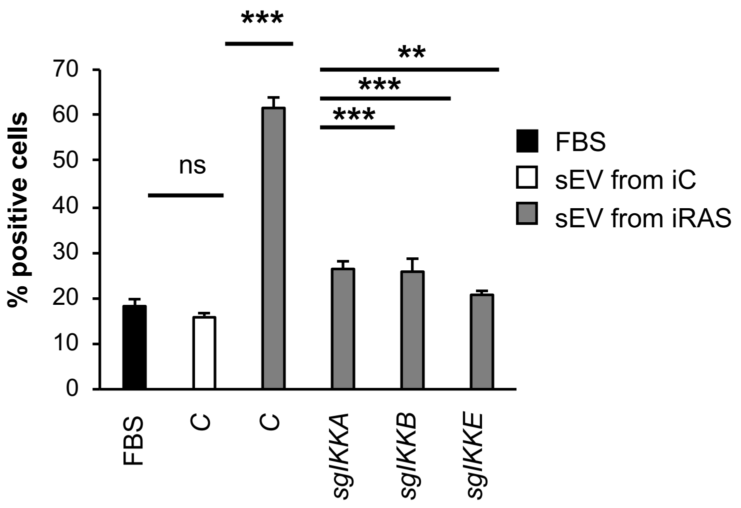

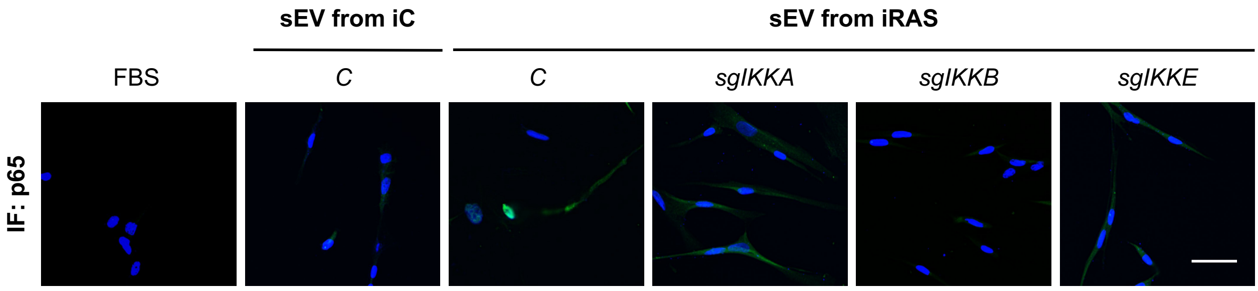

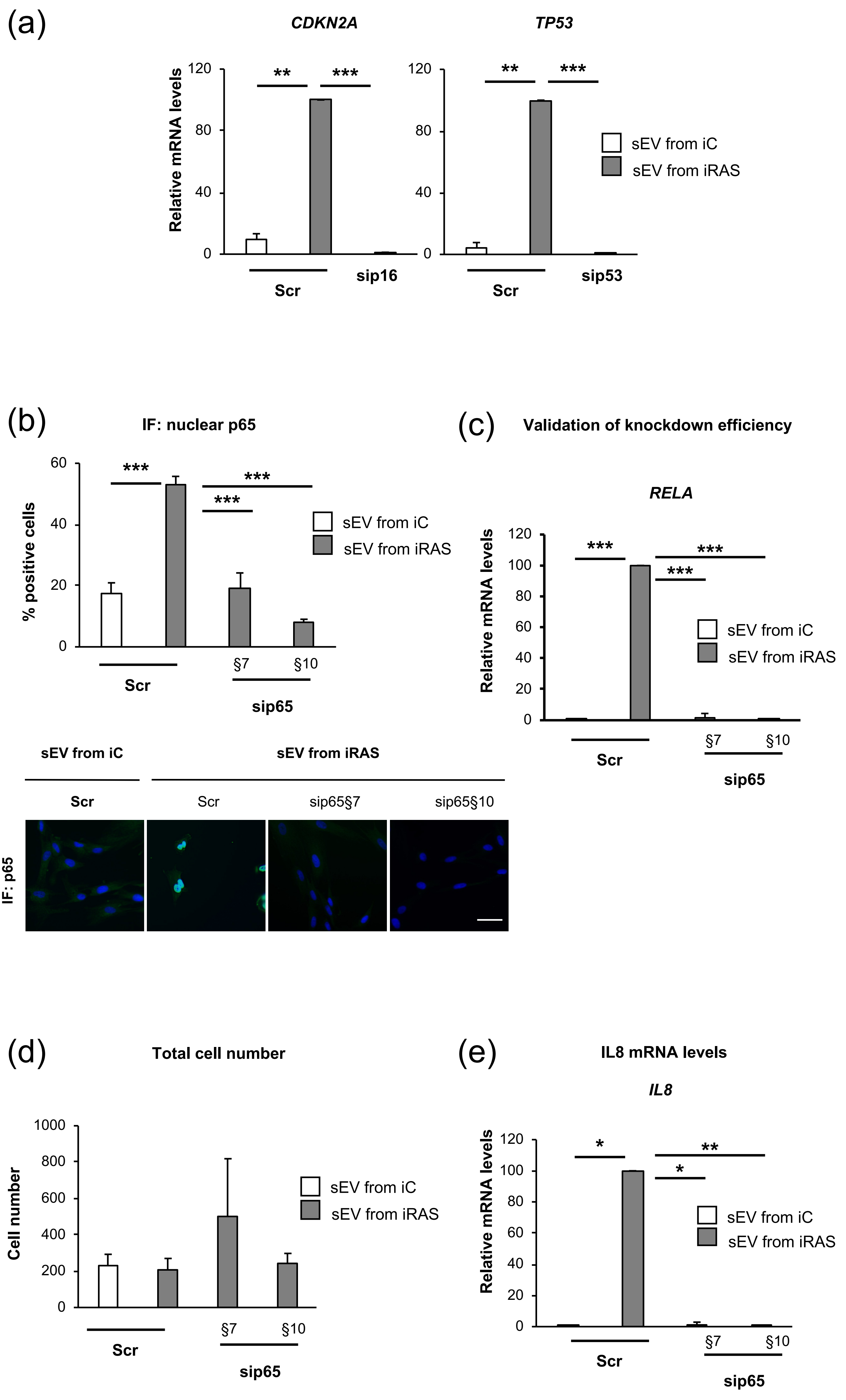
(a)
CDKN2A
TP53
Relative mRNA levels
120
80
40
0
**
***
Scr
sip16
sip53
sEV from iC
sEV from iRAS
(b)
IF: nuclear p65
% positive cells
60
40
20
0
***
§7
§10
Scr
sip65
sEV from iC
sEV from iRAS
Scr
sip65§7
sip65§10
IF: p65
(c)
Validation of knockdown efficiency
RELA
Relative mRNA levels
120
100
80
60
40
20
0
***
§7
§10
Scr
sip65
(d)
Total cell number
Cell number
1000
800
600
400
200
0
§7
§10
Scr
sip65
(e)
IL8 mRNA levels
IL8
Relative mRNA levels
*
**

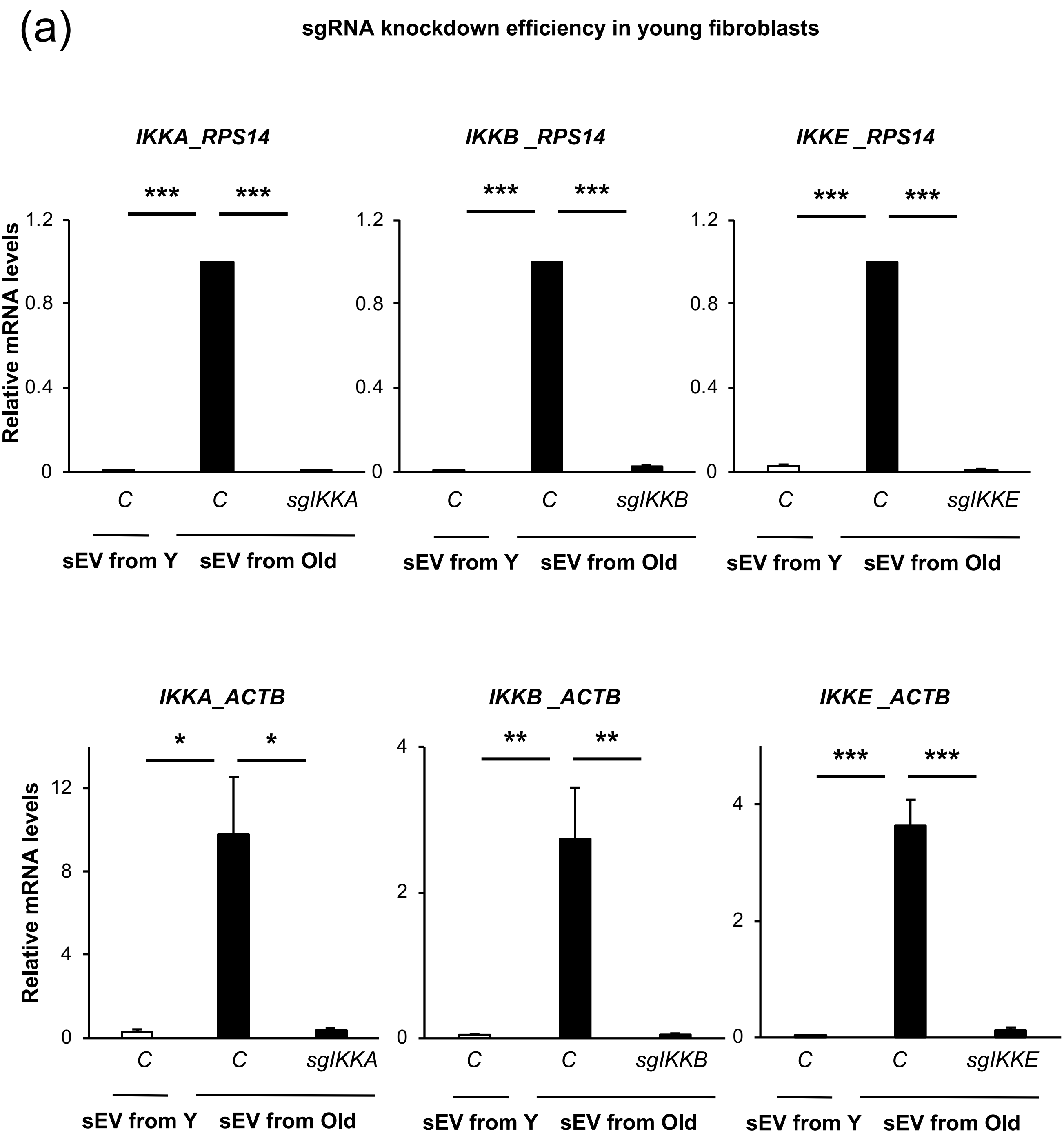
(a)
sgRNA knockdown efficiency in young fibroblasts
IKKA_RPS14
IKKB _RPS14
IKKE _RPS14
Relative mRNA levels
***
*
**
C
sgIKKA
sgIKKB
sgIKKE
sEV from Y
sEV from Old
IKKA_ACTB
IKKB _ACTB
IKKE _ACTB
